# Mechanical Wiping Increases the Efficacy of Liquid Disinfectants on SARS-CoV-2

**DOI:** 10.3389/fmicb.2022.847313

**Published:** 2022-03-22

**Authors:** Angela Sloan, Samantha B. Kasloff, Todd Cutts

**Affiliations:** National Microbiology Laboratory, Applied Biosafety Research Program, Safety and Environmental Services, Public Health Agency of Canada, Winnipeg, MB, Canada

**Keywords:** SARS-CoV-2, biocide, disinfection, fomites, QCT-2, wiping, wipe

## Abstract

High-touch environmental surfaces are acknowledged as potential sources of pathogen transmission, particularly in health care settings where infectious agents may be readily abundant. Methods of disinfecting these surfaces often include direct application of a chemical disinfectant or simply wiping the surface with a disinfectant pre-soaked wipe (DPW). In this study, we examine the ability of four disinfectants, ethanol (EtOH), sodium hypochlorite (NaOCl), chlorine dioxide (ClO_2_), and potassium monopersulfate (KMPS), to inactivate SARS-CoV-2 on a hard, non-porous surface, assessing the effects of concentration and contact time. The efficacy of DPWs to decontaminate carriers spiked with SARS-CoV-2, as well as the transferability of the virus from used DPWs to clean surfaces, is also assessed. Stainless steel carriers inoculated with approximately 6 logs of SARS-CoV-2 prepared in a soil load were disinfected within 5 min through exposure to 66.5% EtOH, 0.5% NaOCl, and 1% KMPS. The addition of mechanical wiping using DPWs impregnated with these biocides rendered the virus inactive almost immediately, with no viral transfer from the used DPW to adjacent surfaces. Carriers treated with 100 ppm of ClO_2_ showed a significant amount of viable virus remaining after 10 min of biocide exposure, while the virus was only completely inactivated after 10 min of treatment with 500 ppm of ClO_2_. Wiping SARS-CoV-2-spiked carriers with DPWs containing either concentration of ClO_2_ for 5 s left significant amounts of viable virus on the carriers. Furthermore, higher titers of infectious virus retained on the ClO_2_-infused DPWs were transferred to uninoculated carriers immediately after wiping. Overall, 66.5% EtOH, 0.5% NaOCl, and 1% KMPS appear to be highly effective biocidal agents against SARS-CoV-2, while ClO_2_ formulations are much less efficacious.

## Introduction

The coronavirus SARS-CoV-2, first discovered in December 2019 in Wuhan City, Hubei province, China, has caused millions of human deaths since its declaration as a pandemic by the World Health Organization in March 2020. Aerosols in close proximity are believed to be the primary mode of transmission (Anderson et al., 2020; Asadi et al., 2020; Morawska and Cao, 2020; World Health Organization [WHO], 2020, 2021; Yang et al., 2021); however, fomite transmission is often overlooked as a method of spread. Fomites are a concern for healthcare settings, where patients shed viral particles on surfaces that may remain viable from hours to weeks depending on the surface type and environmental conditions (i.e., ambient temperature and relative humidity) involved (Chin et al., 2020; Harbourt et al., 2020; Pastorino et al., 2020; Ren et al., 2020; Riddell et al., 2020; van Doremalen et al., 2020). A number of studies have focused on surfaces within health care facilities where patients infected with SARS-CoV-2 are being treated (Zeitler and Rapp, 2014; Chia et al., 2020; Cutts et al., 2020b; Guo et al., 2020; Ong et al., 2020; Wang et al., 2020; Wei et al., 2020; Zhang et al., 2020; Zhou et al., 2020; Zou et al., 2020; Kasloff et al., 2021; Wan et al., 2021) and on high-touch environmental surfaces (HITES; Bloise et al., 2020; Gholipour et al., 2020; Ijaz et al., 2020, 2021; Gavaldà-Mestre et al., 2021; Harvey et al., 2021; Hu et al., 2021; Kozer et al., 2021; Shah et al., 2021). HITES are often decontaminated by applying a suitable disinfectant and allowing it to dry or cleaning with a disinfectant pre-soaked wipe (DPW). Both methods rely on the biocidal nature of the applied chemical; however, the addition of mechanical action combines both inactivation and physical removal of the virus to render a surface “decontaminated” (Sattar and Maillard, 2013). Historically, such sanitation products were not typically tested in a manner recapitulating their use under real-life conditions. Gaps in testing included uncontrolled wiping action, the applied pressure during wiping, and inappropriate contact times (Williams et al., 2007; Sattar and Maillard, 2013; Edwards et al., 2017; Song et al., 2019). Such studies also failed to consider that during wiping, microorganisms could be transferred to adjacent surfaces instead of being removed, depending on the retaining ability of the wipe and the biocidal activity of the disinfectant (Edwards et al., 2017). In response to these shortcomings, ASTM International-E2967-15 (2015) was developed to measure the activity of antimicrobial wipes using a mechanical apparatus: the Wiperator specifically addresses the effect of pressure, cleaning strokes, and time against pathogens on hard non-porous surfaces (ASTM International-E2967-15, 2015).

In this study, we test the ability of DPWs impregnated with one of four microbicidal actives common in the marketplace (Abreu et al., 2013), namely, ethanol (EtOH), sodium hypochlorite (NaOCl), chlorine dioxide (ClO_2_), and potassium monopersulfate (KMPS), to effectively inactivate SARS-CoV-2 using ASTM International-E2967-15 (2015). We also compare the effect of the above disinfectants against dried SARS-CoV-2 in a sit-and-soak assay using ASTM International-E2197-17e1 (2017). This study will provide additional and novel information on the efficacy of these four biocidal agents, in both liquid and DPW forms, on the deadly pathogen, SARS-CoV-2.

## Materials and Methods

### Cells

African green monkey Vero E6 cells (ATCC CRL-1586) were propagated in cell culture medium (CCM) (DMEM; HyClone SH302243.01) supplemented with 10% fetal bovine serum [FBS; Gibco (catalog #) and 1% *v*/*v* penicillin–streptomycin (pen–strep; Gibco LS15140122)]. One day prior to testing, cells were trypsinized (Gibco LS25200056) and seeded into appropriate flasks or plates to reach ∼80% confluence the following day. On the day of testing, the CCM was removed and replaced with virus culture medium (VCM; DMEM + 2% FBS + 10 μl/ml pen/strep) for the duration of the experiment.

### Virus

Virus stocks were prepared as previously described (Cook et al., 2016; Cutts et al., 2020a). Briefly, flasks containing 80% confluent Vero E6 cells were infected with passage 3 stocks of SARS-CoV-2 (*hCoV-19/Canada/ON-VIDO-01/2020, GISAID accession# EPI_ISL_425177*) at an MOI of 0.01. At 3–5 days post infection, the infected supernatant was removed and clarified using low-speed centrifugation at 4,500 × *g* for 10 min. Supernatant was overlaid onto a 20% *w*/*v* sucrose cushion in Tris-NaCl-EDTA buffer (prepared in-house), centrifuged at 28,000 RPM in a Beckman Coulter SW 32 TI rotor for 2 h, and the resulting viral pellet re-suspended in VCM overnight at 4°C. The following day, the pellets were pooled, aliquoted, and quantified as per Reed and Muench (1938). As SARS-CoV-2 is classified as a Risk Group 3 pathogen, all experimental procedures, from inoculum preparation to drying of carriers to disinfectant assays, took place within a Class II B2 BSC in a high-containment laboratory at the National Microbiology Laboratory in Winnipeg, Canada.

### Disinfectants

All four chemical biocides were prepared in accordance with ASTM International-E2197-17e1 (2017). Disinfectants and associated concentrations were chosen based on their potential use in health care facilities, within a laboratory setting, or being a common component in commercial disinfectant formulations.

The disinfectants 66.5% (*v*/*v*) EtOH (Commercial Alcohols P016EA95), 0.5% (*v*/*v*) NaOCl (Imperial Soap and Supplies IMP750-1), and 1% (*w*/*v*) KMPS (Osorno Enterprises Inc., KMPS-1KG-JAR) were prepared in sterile hard water (i.e., containing 0.04% *w*/*v* calcium carbonate). For assays using ClO_2_ (Osorno Enterprises Inc., Power Oxide POT-1L-SET), solutions were prepared 1 day prior to testing and placed at 4°C overnight as per the manufacturer’s instructions. Active chlorine was measured the following morning, and solutions were diluted to 100 ppm (i.e., 0.01%) and 500 ppm (i.e., 0.05%) in hard water for disinfectant assays. Fresh batches of disinfectant were used for each independent experiment.

### Neutralization Assay

A neutralization assay was performed as described previously (Cutts et al., 2019, 2020a) in order to evaluate any interactions between the neutralizers, disinfectants, host cells, and pathogen (Table 1). Combinations of neutralizers and disinfectants were evaluated and are described in the Supplementary Material. Wherever possible, culture medium (VCM) was used as neutralizer for the various experimental assays. However, in quantitative carrier test 2 (QCT-2) assays involving KMPS or ClO_2_, a 1% sodium thiosulfate solution was required to more effectively neutralize the biocides, leading to a more sensitive readout in the reporter assay with the lowest possible limit of detection to assure that any remaining viable virus was detected.

**TABLE 1:** Disinfectants and associated neutralizers used in the present study.

Disinfectant	Manufacturer	Neutralizer (QCT-2 assay)	Neutralizer (Wiperator assay)
66.5% EtOH	Commercial Alcohols	DMEM + 2% (*v*/*v*) FBS + 1% (*v*/*v*) pen–strep	DMEM + 2% (*v*/*v*) FBS + 1% (*v*/*v*) pen–strep
0.5% NaOCl	Imperial Soap and Supplies Ltd.		
1% KMPS	Osorno Enterprises Inc.	1% (*v*/*v*) sodium thiosulfate in hard water	
100 ppm ClO_2_			
500 ppm ClO_2_			

### Quantitative Carrier Test 2 Assay (ASTM 2197-17e1)

The QCT-2 disinfectant efficacy assay was conducted in accordance with ASTM International-E2197-17e1 (2017). Briefly, 170 μl of concentrated SARS-CoV-2 (8.5 logs/ml) was added to a tripartite soil load [12.5 μl of 5% BSA (Sigma A1933), 17.5 μl of 5% tryptone (Sigma T7293), and 50 μl of 4% mucin (Sigma M3895)], used to represent an organic matrix (ASTM International-E2197-17e1, 2017) and simulate the virus in its natural environment (Sattar et al., 2003). Using a positive displacement pipette, 10 μl of inoculum was deposited onto sterile stainless-steel carriers and dried for 1 h within a Class II B2 BSC. Fifty microliters of prepared disinfectant was added to carriers and incubated for 30 s, 1 min, 5 min, or 10 min, after which 950 μl of neutralizer (Table 1) was added and mixed *via* vigorous pipetting. The resulting neutralized solution is herein referred to as the “neat dilution.”

Neat dilutions were 10-fold serially diluted in VCM and, in replicates of five per dilution, added to 96-well plates containing 80% confluent Vero E6 cells for titration. The remaining neat material from each time point was added to 80% confluent Vero E6 cells in a six-well plate to ensure no viable virus remained. All plates were incubated at 37°C for 5 days and any cytopathic effect (CPE) was recorded (Reed and Muench, 1938). A total of three independent experiments with three biological replicates per time point were carried out for each disinfectant.

### Wiperator Assay (ASTM E2967-15)

The effects of mechanical wiping were determined in accordance with the Wiperator methodology, ASTM International-E2967-15 (2015). Inoculum was prepared and deposited onto sterile stainless-steel carriers (“test carriers”) in 10-μl aliquots and dried for 1 h within a Class II B2 BSC. Carriers were placed onto the carrier plate adjacent to non-inoculated secondary carriers (“transfer carriers”) and secured with a magnet on the underside. Sterile J cloths (4 cm × 4 cm) were saturated with 320 μl of freshly prepared biocide and loaded onto the Wiperator Boss (Cutts et al., 2020a). Plates were lifted into place with test carriers subjected to 5 s of automated wiping action at 150 g of pressure. Plates were rotated and transfer carriers subjected to 5 s of wiping with the previously used J cloth. Test and transfer carriers were eluted with 1 ml of the predetermined neutralizer (see Table 1), and the presence of residual viable virus was determined both quantitatively and qualitatively as described for the QCT-2 assay. To determine the additive effect of drying on wiped surfaces, additional test carriers were wiped with each biocide as described; left to air dry for 30 s, 1 min, or 5 min; and subsequently neutralized. No transfer carriers were included for experiments with added drying times. Three independent experiments were conducted for all biocides, with the exception of 100 ppm ClO_2_, which was only assessed in two independent experiments.

## Results

### Quantitative Carrier Test 2 Assay

**FIGURE 1:**
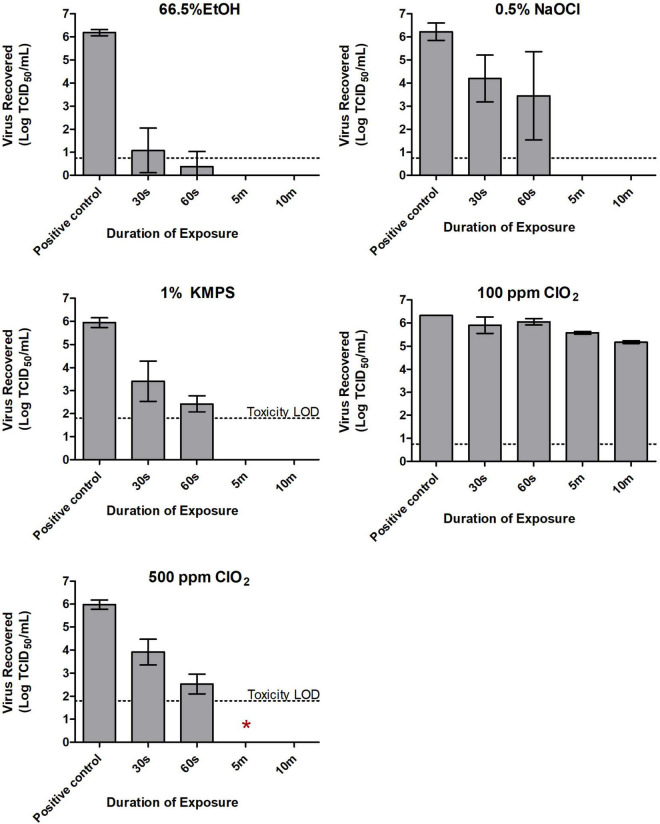
Inactivation of SARS-CoV-2 with 66.5% ethanol (EtOH), 0.5% sodium hypochlorite (NaOCl), 1% potassium monopersulfate (KMPS), 100 ppm chlorine dioxide (ClO_2_), and 500 ppm ClO_2_ during the quantitative carrier test 2 (QCT-2 assay). Inoculated carriers were subjected to various durations of exposure with each of the examined disinfectants. All carriers were neutralized in a pre-determined neutralizer following indicated exposure times, with subsequent viability testing in Vero E6 cells. Dashed lines indicate limits of quantification using the TCID_50_ assay. Toxicity LOD (i.e., limit of detection) reflects residual cytotoxicity in neat dilutions from neutralized carriers. Results represent the means of three independent experiments including three biological replicates each. Symbol *Indicates presence of viable virus in safety testing of a single biological replicate from the indicated treatment group.

When exposed to 66.5% EtOH, the titer of the dried inoculum (6.19 logs) decreased by 5.12 logs/carrier to 1.07 after 30 s of exposure. By 60 s, titers fell below the limit of quantitation (Figure 1), although one of nine wells showed CPE during safety testing (Table 2). Treatment with 0.5% NaOCl resulted in a log decrease of only 2.03 logs/carrier after 30 s and 3.45 logs after 1 min from an initial titer of 6.22 logs/carrier. It was only at the 5-min time point that the virus was completely inactivated by either EtOH or NaOCl in both the TCID_50_ assays (Figure 1) and safety tests (Table 2).

**TABLE 2:** Number of tissue culture-treated wells showing a cytopathic effect following treatment with disinfectants and disinfectant pre-soaked wipes during safety testing during the QCT-2 and Wiperator assays, respectively.

Disinfectant	Virus recovered (log TCID_50_/ml) in dried inoculum	CPE-positive samples in safety tests			
		
		Biocide exposure time			
		
		30 s	1 min	5 min	10 min
QCT-2 assay					
66.5% EtOH	6.19 ± 0.13	3/9	1/9	0/9	–
0.5% NaOCl	6.22 ± 0.38	9/9	9/9	0/9	0/9
500 ppm ClO_2_	5.98 ± 0.20	8/9	5/9	1/9	0/9
100 ppm ClO_2_	6.32 ± 0.00	6/6	6/6	6/6	6/6
1% KMPS	5.95 ± 0.21	9/9	9/9	0/9	0/9

**Disinfectant**	**Virus recovered (log TCID_50_/mL) in dried inoculum**	**CPE-positive samples in safety tests**			
		
		**Drying time post-exposure**			
		
		**30 s**	**1 min**	**5 min**	**10 min**

Wiperator assay					
65% EtOH	6.32 ± 0.13	0/9	0/9	0/9	0/9
0.5% NaOCl	6.21 ± 0.21	0/9	0/9	0/9	0/9
500 ppm ClO_2_	6.05 ± 0.10	6/9	6/9	5/9	4/9
100 ppm ClO_2_	6.32 ± 0.00	6/6	6/6	6/6	6/6
1% KMPS	5.96 ± 0.24	1/9	0/9	0/9	0/9

*–, not measured.*

Exposing SARS-CoV-2 to ClO_2_ at a concentration of 100 ppm had a negligible effect on virus viability. A contact time of 30 s and 1 min with the disinfectant resulted in less than 0.5 log loss of viable virus (Figure 1). Even with 10 min of contact, only a 1.15 log decrease in viral titer was recorded (Figure 1), with all wells showing CPE in safety tests (Table 2). As such, only two independent experiments were carried out at this concentration. Increasing the concentration of ClO_2_ to 500 ppm produced more favorable results. An initial concentration of 5.98 logs of dried SARS-CoV-2 was reduced to 3.91 logs/carrier after a 30-s exposure (Figure 1). By 60 s, 3.45 logs of viable virus were recovered, and by 5 min, no detectable virus remained on the carrier surface in TCID_50_ assays (Figure 1). However, one of nine biological replicates (i.e., a single carrier from only one of three independent experiments) showed CPE in safety tests at the 5-min mark (Table 2). Increasing contact time to 10 min left no viable virus detectable in either the TCID_50_ assay (Figure 1) or safety test (Table 2).

Following exposure of 1% KMPS to the inoculated carriers, a 2.54 log decrease in viable virus from 5.95 to 3.41 logs/carrier occurred within 30 s and a 3.52 log decrease to 2.43 logs/carrier occurred by 1 min (Figure 1), with all nine safety test wells showing CPE (Table 2). After 5 min, no detectable virus remained in TCID_50_ assays (Figure 1) and safety tests (Table 2).

### Wiperator Assay

**FIGURE 2:**
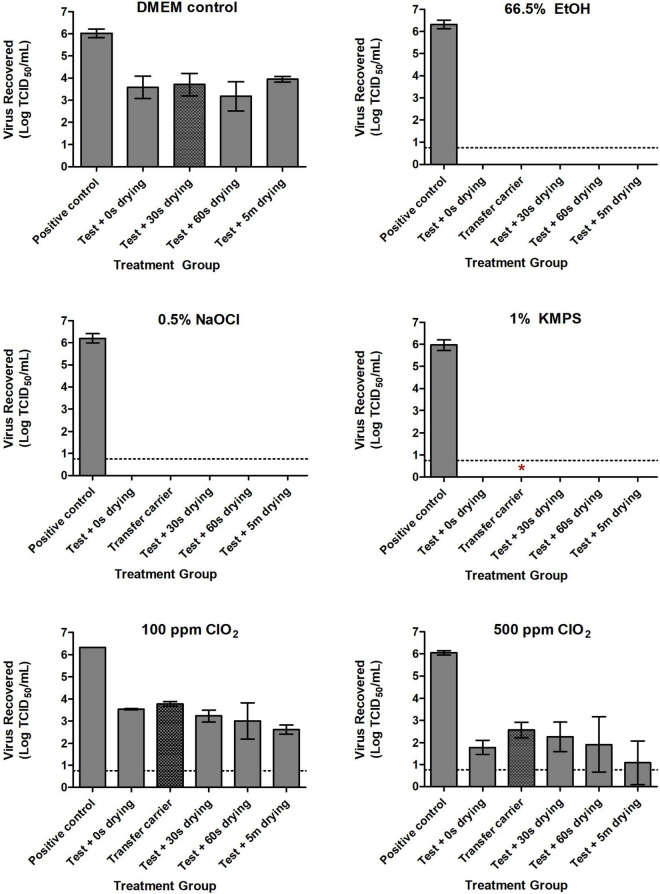
Inactivation of SARS-CoV-2 with Dulbecco’s Modified Eagle Medium (DMEM; positive control), 66.5% ethanol (EtOH), 0.5% sodium hypochlorite (NaOCl), 1% potassium monopersulfate (KMPS), 100 ppm chlorine dioxide (ClO_2_), and 500 ppm ClO_2_ during the Wiperator assay. Inoculated carriers were subjected to 5 s of wiping with disinfectant pre-soaked wipes infused with each of the examined disinfectants. Sterile uninoculated “transfer” carriers demonstrate transfer potential of viable virus to a clean surface through a used disinfectant presoaked wipe. All carriers were neutralized in 1 ml of a pre-determined neutralizer following indicated treatments, with subsequent viability testing in Vero E6 cells. Dashed lines indicate limits of quantification using the TCID_50_ assay. Results represent the means of three independent experiments including three biological replicates each. Symbol *Indicates presence of viable virus in safety testing of a single biological replicate from the indicated treatment group.

Incorporating the examined disinfectants into a mechanical wipe markedly reduced the time required to inactivate SARS-CoV-2. With 66.5% EtOH, 0.5% NaOCl, and 1% KMPS, no viable virus remained on inoculated test carriers after 5 s of wiping, nor did any remain on secondary transfer carrier after 5 s of wiping with the used DPW (Figure 2). Infectious virus was recovered, however, on both test and transfer carriers after treatment with the ClO_2_-impregnated DPWs. Not surprisingly, more virus remained on test carriers treated with the lower concentration of disinfectant, with 3.54 and 1.78 logs of SARS-CoV-2 recovered after treatment with DPWs containing 100 and 500 ppm ClO_2_, respectively (Figure 2). Notably, the amount of viable virus declined only slightly after the inoculated carriers were dried for increasingly longer durations post-wiping. Safety testing of the remaining neutralized viral solutions showed no viable virus for carriers treated with 66.5% EtOH or 0.5% NaOCl (Figure 2). However, a single biological replicate wiped with 1% KMPS resulted positive in safety testing, while the majority of wells were positive when using ClO_2_ (Table 2).

## Discussion

The ability of SARS-CoV-2 to remain viable on non-porous surfaces for days to weeks, depending on environmental conditions, has been well documented (Chin et al., 2020; Harbourt et al., 2020; Pastorino et al., 2020; Ren et al., 2020; Riddell et al., 2020; van Doremalen et al., 2020). This observation poses significant implications for HITES and healthcare settings where the pathogen may be abundant due to congregation and prolonged stay of infirm patients within a confined tempered space. As a generalization, enveloped viruses, including SARS-CoV-2, are more susceptible to environmental factors that non-enveloped viruses, as their phospholipid envelope is sensitive to ambient conditions such as heat, desiccation, detergents, and chemical degradation (Navaratnarajah et al., 2008; Lin et al., 2020). In this study, rather than exploiting this sensitivity, we attempt to inactivate SARS-CoV-2 as it would be found on environmental surfaces [i.e., within a protective organic matrix (Sattar et al., 2003; Terpstra et al., 2007)] using four chemical biocides, EtOH, NaOCl, KMPS, and ClO_2_, alone or with the addition of a mechanical wipe.

Several studies have examined the ability of DPWs, with or without incorporation of the Wiperator, to effectively decontaminate inoculated surfaces (Sattar et al., 2003; Williams et al., 2009; Sattar and Maillard, 2013; Lopez et al., 2014; ASTM International-E2967-15, 2015; Ramm et al., 2015; Becker et al., 2019; Song et al., 2019; Cutts et al., 2020a,^2021^). However, studies examining the effect of EtOH and NaOCl on SARS-CoV-2 have only examined their ability to inactivate the virus in liquid suspension for prolonged contact times (Chin et al., 2020). Additional inactivation studies involving these two biocides and human coronaviruses do exist; however, those involving EtOH have typically only examined various hand sanitizer formulations (Kratzel et al., 2020), while research involving NaOCl has been limited to coronaviruses existing prior to the emergence of SARS-CoV-2 (Kampf et al., 2020; Janik et al., 2021). Furthermore, the direct effects of KMPS and ClO_2_ on SARS-CoV-2 have yet to be reported *in vitro*, although both agents have displayed notable antimicrobial activity against a wide array of pathogens (Su and D’Souza, 2012; Hinenoya et al., 2015; Morin et al., 2015; Conlon-Bingham et al., 2016; Praeger et al., 2018; Sonthipet et al., 2018).

For the QCT-2 assay, the inactivation of SARS-CoV-2 upon exposure to the different disinfectants under analysis was variable. Viral titer showed the steepest decline once exposed to 66.5% EtOH, with a 5.12 log reduction after only 30 s and the majority of remaining virus below the limit of quantification after 60 s. Considering the disinfectant’s widespread incorporation into hand sanitizers targeting SARS-CoV-2 (Kratzel et al., 2020), its efficacy against other human coronaviruses (Kampf et al., 2020; Janik et al., 2021), and the widespread antimicrobial activity of alcohols in general (McDonnell and Russell, 1999; Guthery et al., 2005), these findings are not surprising. The virus displayed a more gradual inactivation profile while exposed to 0.5% NaOCl, with all viable virus inactivated by the 5-min mark. As with all of the disinfectants tested in this study, no additional time points were examined between 1 and 5 min of biocide exposure and, consequently, more precise times-to-inactivation within this range could not be determined. Interestingly, in a study of mouse hepatitis virus (MHV), a proposed potential surrogate for SARS-CoV-1, a 30-s exposure to 0.21% NaOCl inactivated >6 logs of challenge virus when dried on a non-porous surface to below the limit of detection (a 4.4-log demonstrable loss) (Dellanno et al., 2009). The use of an organic soil load in our study was likely a significant contributor to the extended exposure time required to inactivate SARS-CoV-2, despite the NaOCl concentration being 2× higher. Additionally, it is possible that differences between these coronaviruses are sufficient enough to alter their susceptibility to NaOCl. This has been seen in other closely related viruses, and it has been suggested that even minor changes in their viral composition can result in substantial differences in their inactivation kinetics (Sigstam et al., 2013; Ge et al., 2021).

Like with 66.5% EtOH and 0.5% NaOCl, complete inactivation of nearly 6 logs of dried SARS-CoV-2 was achieved after a 5-min exposure to 1% KMPS on stainless steel during the QCT-2 assay. Notably, as with 66.5% EtOH, the majority of virus (i.e., 4.25 logs) was degraded within the first minute of contact with the biocide, with full inactivation by the 5-min mark. KMPS is widely used as a chlorine-free oxidizing agent and is the main active ingredient in the multi-purpose laboratory disinfectant, Virkon (Cleanroom Technology, 2020). Virkon is known to have a wide range of antimicrobial activity against viruses, bacteria, and fungi (Gasparini et al., 1995; LANXESS, 2017) and has proven to be effective against SARS-CoV-2 (Syndel) Syndel (2019). It is noteworthy, however, that the KMPS ingredient by itself can inactivate the virus without the other reagents in Virkon (i.e., the cleaning agent sodium dodecylbenzenesulfonate and the detergent sulfamic acid), which are seemingly essential for its biocidal action.

The use of ClO_2_ has been shown to inactivate a variety of viruses, including SARS-CoV-2, in wastewater, albeit in conjunction with several other antimicrobial agents at low concentrations (Ge et al., 2021; Singh et al., 2021). Furthermore, ClO_2_ was shown to effectively inactivate SARS-CoV-1 after 30 min of exposure at a dosage of only 40 ppm (Wang et al., 2005). More recently, researchers inactivated 5 logs of SARS-CoV-2 using pure ClO_2_ at 80 ppm against SARS-CoV-2 in a suspension for as little as 10 s (Hatanaka et al., 2021). Our study followed the ASTM 2197-17 standard and showed that ClO_2_ at a lower concentration of 100 ppm did not fare as effectively against SARS-CoV-2 when dried on a hard non-porous surface, with only a 1.39-log reduction after a full 10 min of exposure. While others inactivated SARS-CoV-2 at lower concentrations of ClO_2_, those studies used a suspension test, had reduced protein content, used greater volumes of the ClO_2_, and in some cases had less virus. Our study format is a more challenging approach than a suspension test as it uses high titers of virus incorporating a higher protein content along with a mucin protectorate and uses lower amount of ClO_2_. These factors illustrate the importance of comparing efficiencies against biocides and their practical use under real-world conditions.

The effect of combining microbicidal actives and mechanical wiping action on the inactivation of viruses and bacteria has been evaluated in several studies (Tebbutt, 1988; Threlkeld et al., 1993; Williams et al., 2009; Abreu et al., 2013; Lopez et al., 2014; ASTM International-E2967-15, 2015; Ramm et al., 2015; Sattar et al., 2015; Edwards et al., 2017; Becker et al., 2019; Song et al., 2019; Cutts et al., 2020a). The benefits of mechanical action include its ability to remove the organic debris that could hinder the biocidal action of the disinfectant (Song et al., 2019), as well as dislodging the infectious material from its dried state to allow optimal penetration by the disinfectant. Here, we impregnated J Cloths with each of four disinfectants and employed the Wiperator to apply a continuous controlled wiping action on stainless steel carriers inoculated with SARS-CoV-2. Remarkably, after only 5 s of wiping with either 66.5% EtOH, 0.5% NaOCl, or 1% KMPS, no viable virus remained on any of the inoculated test carriers. The viral inactivation time for these three biocides during the QCT-2 assay was 5 min, signifying that the incorporated wiping action greatly increased the disinfectants’ efficacy. Furthermore, no quantifiable virus was recovered from transfer carriers after a 5-s wipe with used DPWs infused with these biocides. These data are similar to other studies assessing the wiping action of DPWs on viruses. Cutts et al. (2020a) demonstrated a complete inactivation and >6 log reduction of Ebola virus and vesicular stomatitis virus, respectively, on inoculated test carriers with no transfer of infectious virus to secondary carriers after 15 s of wiping with accelerated hydrogen peroxide (AHP)- or quaternary ammonium compound (QAC)-impregnated wipes. Moreover, Threlkeld et al. (1993) showed that wiping with DPWs infused with either isopropyl alcohol, hydrogen peroxide (H_2_O_2_), or iodophor for 5 s removed adenovirus 8 from Goldmann tonometer and pneumotonometer tips, while a 5-min submersion time was required for inactivation by the same compounds in the absence of wiping.

Although SARS-CoV-2 was resistant to degradation by 100 ppm ClO_2_, it was inactivated using 500 ppm (0.05%) ClO_2_ by the 5-min time point during the QCT-2 assay. Notably, the virus was far more susceptible to both concentrations of the biocide when wiping action was implemented. After 5 s of wiping with 100 ppm (0.01%) and 500 ppm ClO_2_-infused wipes, a 2.78 and 4.27 log reduction in viable virus was observed on inoculated test carriers, respectively. Considering that both concentrations of the disinfectant had negligible-to-minor effects on the virus during the QCT-2 assay (a 0.42 and 2.07 log reduction after 30 s of exposure to 100 and 500 ppm, respectively), these findings provide further support for the enhancement of viral inactivation when mechanical wiping is incorporated in the disinfection process.

Interestingly, in Wiperator tests involving ClO_2_, considerably high amounts of viable virus were recovered from both test and transfer carriers, indicating that transferring an infectious material from one surface to another *via* wiping is a legitimate concern. As such, it is imperative that any disinfection strategy involve the use of biocides proven effective against the infectious agent of concern rather than simply relying on commercial products due to ease or availability.

As seen in other studies, many factors contribute to the efficacy of antimicrobial disinfection, including the pathogen type (e.g., bacteria, viruses, spores, fungi, etc.), presence of an organic matrix, disinfectant used (e.g., type and concentration), and duration of exposure to the disinfectant (Sattar et al., 2003, 2015; Terpstra et al., 2007; Williams et al., 2009; Sattar and Maillard, 2013; Lopez et al., 2014; ASTM International-E2967-15, 2015; Ramm et al., 2015; Becker et al., 2019; Song et al., 2019; Cutts et al., 2020a; Ijaz et al., 2020). We have shown here that SARS-CoV-2 is most quickly inactivated by 66.5% EtOH, followed by 1% KMPS, 0.5% NaOCl, and then ClO_2_. Even when dried within a protective organic matrix, roughly 6 logs of virus can be inactivated using the three former biocides within 5 min of exposure, while 500 ppm ClO_2_ requires a longer duration (at least 10 min). We have also demonstrated that incorporating these biocides into a mechanical wipe greatly enhances their efficacy against the virus, and in agreement with other studies (Tebbutt, 1988; Ramm et al., 2015; Cutts et al., 2020a), infectious agents can be transferred to adjacent surfaces through wiping with a DPW if impregnated with an ineffective biocide. Nevertheless, wiping surfaces with DPWs infused with either 66.5% EtOH, 0.5% NaOCl, or 1% KMPS should be considered an effective means of inactivating viable SARS-CoV-2.

Although this study was on SARS-CoV-2 isolate Wuhan-Hu-1, we believe that the present findings would also apply to other circulating variants. Others have observed that there was no difference between SARS-CoV-1 and SARS-CoV-2 deposited on surfaces (van Doremalen et al., 2020) or in aerosol survival (Smither et al., 2020). Furthermore, other investigators have looked at the effect of biocides such as alcohol on variants and no difference was reported (Meister et al., 2021).

## Conclusion

Here, we evaluated readily available disinfectants or components of formulated disinfectants that can be used within healthcare facilities or by the general public, either on their own or by incorporating wiping. Following simple application, ethanol was the most effective at reducing 6 logs of dried virus within a soil load in a short amount of time, while sodium hypochlorite (bleach) and KMPS required longer contact times. By incorporating a mechanical wipe, the virucidal effects of ethanol, sodium hypochlorite, and KMPS were immediate, with no detectable virus remaining after only 5 s of wiping the surface.

## Data Availability Statement

The original contributions presented in the study are included in the article/Supplementary Material, further inquiries can be directed to the corresponding author.

## Author Contributions

SK and TC contributed to the conception and design of the study and performed the methods. AS drafted the manuscript. All authors analyzed the results, contributed to the article, and approved the submitted version.

## Conflict of Interest

The authors declare that the research was conducted in the absence of any commercial or financial relationships that could be construed as a potential conflict of interest.

## Publisher’s Note

All claims expressed in this article are solely those of the authors and do not necessarily represent those of their affiliated organizations, or those of the publisher, the editors and the reviewers. Any product that may be evaluated in this article, or claim that may be made by its manufacturer, is not guaranteed or endorsed by the publisher.

## Abbreviations

ClO_2_: chlorine dioxide
DPW: disinfectant pre-soaked wipe
EtOH: ethanol
HITES: high-touch environmental surfaces
KMPS: potassium monopersulfate
NaOCl: sodium hypochlorite.
