# Supplementary material for: Mechanical Wiping Increases the Efficacy of Liquid Disinfectants on SARS-CoV-2
Source: Front Microbiol. 2022 Mar 22;13:847313. doi: 10.3389/fmicb.2022.847313 (PMC8981239; doi:10.3389/fmicb.2022.847313)
Supplement: Supplementary file 1 [file Data_Sheet_1.docx]

**Supplementary Material**

**Methods: Neutralization Assay**

On the day of neutralization assays, VCM and 1% sodium thiosulfate were prepared fresh and a low titer virus stock (~100–200 TCID_50_ units per mL) of SARS CoV-2 (hCOV-19/Canada/ON-VIDO-01/2020, GISAID accession# EPI_ISL_425177) utilized. All neutralization controls were performed in triplicate over a single experiment.

VCM (Negative Control)

In 96-well and 6-well plates, Vero E6 cells were cultured in VCM and used as a control for evaluation of cytotoxicity.

Neutralizer (Cytotoxicity Control)

Each neutralizer was ten-fold serially diluted from 10^0^ (neat) to 10^-3^ in VCM and 50 µL added to 96-well plates containing Vero E6 cells (i.e. reporter cells) in replicates of five. The remaining material was added to a 6-well plate containing reporter cells in 3 mL of VCM for safety testing. Five days post-exposure, cells were scored for cytotoxicity.

Neutralizer + Disinfectant (Cytotoxicity Control)

For the QCT-2 method, 50 µL of prepared biocides was added to 950 µL of prepared neutralizer. Solutions were ten-fold serially diluted (10^0^ to 10^-3^) and 50 µL added to reporter cells containing 150 µL of VCM in replicates of five. Five days post-exposure, the cells were scored for cytotoxicity.

For the Wiperator assay, a 4 x 4 cm J cloth wipe containing 320 µL biocide was fitted into the Wiperator apparatus, after which a sterile stainless steel carrier was wiped for 5 seconds and placed into 1 mL of neutralizer. The solution was mixed by pipetting and ten-fold serially diluted in VCM (10^0^ to 10^-3^). In replicates of five per dilution, 50 µL were added to an 80% confluent monolayer of reporter cells. Any remaining material was added to a 6-well plate containing reporter cells in 3 mL of VCM for safety testing. Five days post-exposure, the cells in both the 96-well and 6-well plates were scored for cytotoxicity.

Virus (Positive/CPE Control)

For the QCT-2 assay, SARS-CoV-2 was prepared to 4-5 logs/mL, and 10 µL of virus added to 990 µL of VCM. The positive control was ten-fold serially diluted in VCM and, in replicates of five from 10^0^ (neat) to 10^−3^, 50 µL of each dilution was added to 96-well reporter plates containing Vero E6 cells in 150 µL of VCM. Cells were scored for CPE 5 days post-inoculation for viral titration as per Reed and Muench (Reed and Muench, 1938).

For safety testing, the same steps were followed as above. Exceptionally, the virus was diluted to 2 logs/mL and 10 µL (2-6 TCID_50_ units per well) added to 6-well plates containing reporter cells. This test was performed to ensure that a minimal amount of virus was sufficient to infect the cells. Five days post-exposure, 6-well plates were qualitatively scored for CPE.

Neutralizer + Virus (Positive/CPE Control)

For the QCT-2 assay, SARS-CoV-2 was prepared to 4-5 logs/mL, and 10 µL of virus added to 990 µL of neutralizer. Solutions were ten-fold serially diluted in VCM and, in replicates of five for each dilution (10^0^ to 10^−3^), 50 µL of diluted virus added to 96-well plates containing Vero E6 cells in 150 µL of VCM. Cells were scored for viral CPE 5 days post-inoculation as per Reed and Muench (Reed and Muench, 1938).

For the Wiperator assay, a 4 x 4 cm J cloth containing 320 µL of biocide was fitted into the Wiperator apparatus. A sterile stainless steel carrier was then wiped for 5 seconds and placed into 1 mL of neutralizer. The solution was mixed by pipetting and 10 µL of SARS-Cov-2 (prepared to 4-5 logs TCID_50_/mL) was added, ten-fold serially diluted in VCM and, in replicates of five for each dilution (10^0^ to 10^−3^), 50 µL of diluted virus added to 96-well plates containing Vero E6 cells in 150 µL of VCM. Cells were scored for viral CPE 5 days post-inoculation as per Reed and Muench (Reed and Muench, 1938).

For safety testing, the same steps were followed as above. Exceptionally, the virus was diluted to 2 logs/mL and 10 µL (2-6 TCID_50_ units per well) was added to 6-well plates containing Vero E6 cells in 3 mL of VCM. This test was performed to ensure that a minimal amount of virus was enough to infect the cells. Five days post-exposure, 6-well plates were qualitatively scored for CPE.

Neutralizer + Disinfectant + Virus (Positive/CPE Control)

For the QCT-2 assay, 50 µL of biocide was added to 950 µL of VCM and SARS-CoV-2 prepared to 4-5 logs/mL. Ten 10 µL of virus was added to the neutralized solution, which was then incubated for 10 minutes at room temperature and ten-fold serially diluted from 10^0^ to 10^-3^ in VCM. In replicates of five for each dilution, 50 µL of the diluted virus was subsequently added to 96-well plates containing 150 µL of VCM. Cells were scored for viral CPE 5 days post-inoculation as per Reed and Muench (Reed and Muench, 1938).

For the Wiperator assay, a 4 x 4 cm J cloth containing 320 µL of biocide was fitted into the Wiperator apparatus. A sterile stainless steel carrier was then wiped for 5 seconds and placed into 1 mL of neutralizer. SARS-CoV-2 was prepared to 4-5 logs/mL, after which 10 µL of the virus was added to the solution and ten-fold serially diluted in VCM. In replicates of five for each dilution (10^0^ to 10^-3^), 50 µL of the diluted virus was added to 96-well plates containing Vero E6 cells in 150 µL of VCM. Cells were scored for viral CPE 5 days post-inoculation as per Reed and Muench (Reed and Muench, 1938).

For safety testing, the same steps were followed as above. Exceptionally, the virus was diluted to 2 logs/mL and 10 µL (2-6 TCID_50_ units per well) added to 6-well plates containing Vero E6 cells in 3 mL of VCM. This test was performed to ensure that a very minimum amount of virus was enough to infect the cells. Five days post-exposure, 6-well plates were qualitatively scored for CPE.

**Results: Neutralization Assay**

For QCT-2 assays, a volume of 950 μL of VCM was sufficient to neutralize 50 μL of EtOH and NaOCl, with no evident cytotoxicity even at the neat dilution in cell culture. A 1% sodium thiosulfate neutralizer was required to mitigate the cytotoxic effects of KMPS and ClO_2_ (data not shown), though with 500 ppm ClO2, a degree of cytotoxicity remained at the neat dilution. For assays involving the Wiperator, VCM was used as a diluent against all biocides as no negative effects were observed against the virus or cells with residual biocidal agent on the stainless steel carriers (data not shown).
